# Subcutaneous Emphysema and Severe Interstitial Lung Disease in the Setting of Anti-MDA 5 Positive Dermatomyositis in a Hispanic Patient

**DOI:** 10.1155/crrh/2017703

**Published:** 2025-01-23

**Authors:** Sweta Subhadarshani, Brad Woodie, Emerson Bookal, Justin Reed

**Affiliations:** ^1^Department of Dermatology, Perelman School of Medicine, University of Pennsylvania, 3400 Civic Center Boulevard, South Pavillion-1st Floor, Philadelphia 19104, Pennsylvania, USA; ^2^College of Medicine, University of Cincinnati College of Medicine, University of Cincinnati, Cincinnati, Ohio, USA; ^3^Department of Internal Medicine, Ocala Regional Medical Center, UCF, Ocala, Florida, USA

**Keywords:** anti-MDA5 dermatomyositis, dermatology, interstitial lung disease, rheumatology

## Abstract

Antimelanoma differentiation-associated gene 5 (MDA5) dermatomyositis (DM) is a subtype of DM associated with characteristic mucocutaneous features. These individuals have an increased risk of developing interstitial lung disease (ILD) that ultimately leads to a complicated clinical course. Certain clinical findings suggest anti-MDA5 positive DM over anti-MDA5 negative DM, including cutaneous ulcers, diffuse nonscarring alopecia, and panniculitis. ILD and pneumomediastinum are known to be two of the most important pulmonary complications of anti-MDA5 DM because of the possibility of a rapidly progressive course and poor survival. This case outlines the unique presentation of pneumomediastinum, subcutaneous emphysema, and ILD in a patient with anti-MDA5 positive DM.

## 1. Introduction

Antimelanoma differentiation-associated gene 5 (MDA5) dermatomyositis (DM) is a rare subtype of idiopathic inflammatory myositis that presents distinct clinical challenges. Anti-MDA5 DM may be difficult to detect, as it is often hypomyopathic or amyopathic [1, 2]. Cutaneous manifestations of DM may also be subtle, especially in skin of color. Early recognition of anti-MDA5 DM is important due to the risk of rapidly progressive and potentially fatal interstitial lung disease (ILD) [2]. We present a case of anti-MDA5 DM in a Hispanic woman who presented with progressive ILD, pneumomediastinum, and associated subcutaneous emphysema in the setting of nonspecific myopathy and characteristic skin changes.

## 2. Case Presentation

A 71-year-old Hispanic woman with a past medical history of atrial fibrillation, chronic obstructive pulmonary disease, and type 2 diabetes mellitus presents to the emergency department with complaints of progressively worsening facial and chest swelling. The swelling was accompanied by a dry cough and shortness of breath that worsened when lying flat. Of note, she was being managed by an outside facility for “nonspecific myopathy” and “eyelid dermatitis” with azathioprine and prednisone 20 mg.

**Figure 1:**
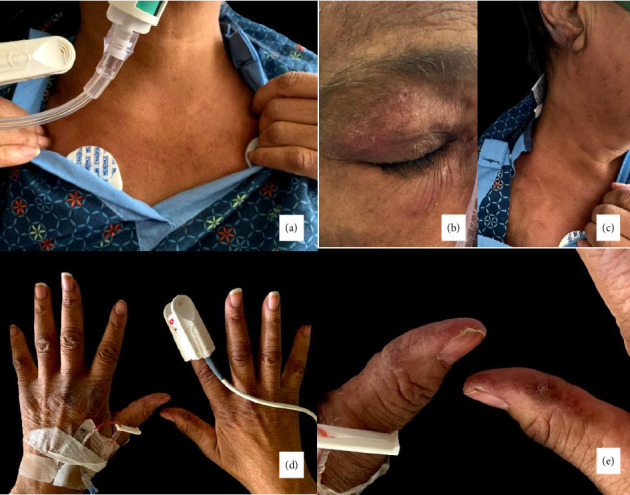
(a) Poikilodermatous changes over the chest (V-sign). (b) Skin-colored papules over interphalangeal joints (Gottron's sign). (c) Significant swelling of the face and neck with crepitus. (d-e) Hyperkeratosis of margins of fingers and thumb (mechanic's hands).

She was hypoxemic, requiring high flow oxygen to maintain a saturation of more than 94%. The mucocutaneous examination was remarkable for poikilodermatous changes over the chest (V-sign) (Figure 1(a)), atrophy, telangiectasia, and a heliotrope rash (Figure 1(b)), skin-colored papules over interphalangeal joints (Gottron's sign) (Figures 1(d) and 1(e)), and hyperkeratosis of margins of fingers and thumb (mechanic's hands) (Figures 1(d) and 1(e)). In addition, there was significant swelling of the face and neck with crepitus (Figure 1(c)).

**Figure 2:**
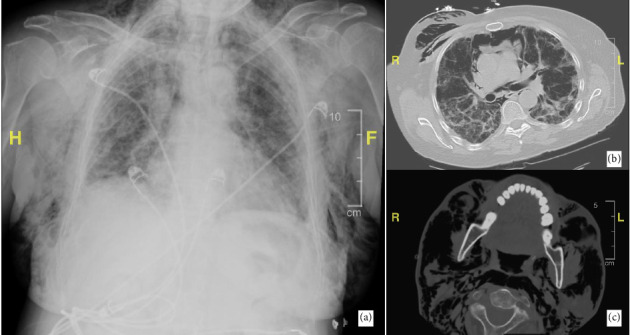
(a) Diffuse subcutaneous emphysema with bilateral infiltrates on chest x-ray. (b) CT chest showing subcutaneous emphysema and pneumomediastinum with associated lung infiltrates and areas of scarring bilaterally. (c) CT neck showing subcutaneous emphysema involving the neck with extension into the head.

Chest x-ray and CT chest/neck revealed ground glass opacities, interstitial infiltrates, moderate to severe pneumomediastinum, and extensive subcutaneous emphysema in the upper chest with extension into the neck and head (Figure 2). Laboratory evaluation was remarkable for a positive anti-MDA 5 antibody, marginally elevated aldolase (8.6 U/L), and normal creatinine kinase. Muscle MRI showed minimal inflammation. A punch biopsy from the skin revealed vacuolar interface dermatitis with increased mucin. A diagnosis of anti-MDA-5 positive DM was made on clinicopathological correlation.

The patient's cough continued to worsen during the hospital course. She underwent a bronchoscopy with no significant findings. She was started on a high-dose steroid regimen of methylprednisolone 500 mg twice daily for 3 days followed by a tapering dose of prednisone and azathioprine.

The patient experienced a decrease in oxygen requirements and was able to maintain saturation above 94% on 3L. The patient was being managed in a community hospital setting and we did not have an inpatient dermatologist/rheumatologist or resources for intravenous immunoglobulin (IVIG) infusions. She was discharged with a recommendation for close follow-up with a rheumatologist and further treatment at a tertiary care center.

## 3. Discussion

Anti-MDA5 DM is a rare yet clinically significant entity due to the risk of fatal pulmonary complications. We describe a case of anti-MDA5 DM presenting to a community hospital with rapidly progressive ILD, pneumomediastinum with extensive subcutaneous emphysema, and subtle cutaneous findings that improved with high-dose glucocorticoids and azathioprine.

In patients with DM, the presence of anti-MDA5 leads to an 18-fold increase in the risk for ILD [3]. MDA5 antibodies are specific for DM and are not found in other connective tissue disorders or inflammatory myopathies [3]. ILD may progress rapidly, as in the current case, and the mortality rate of rapidly progressive ILD in anti-MDA5 DM has been estimated at 80% [2]. Spontaneous pneumomediastinum may arise from alveolar air leakage that dissects along pulmonary vessels [4] and severe cases can require rescue lung transplantation [5]. In the present case, the patient's extensive subcutaneous emphysema (contiguous with pneumomediastinum) led to visible swelling of the face and chest.

Early diagnosis is critical when severe ILD is present, but recognition can be challenging. Anti-MDA5 DM was first described in Japanese patients [6], and much of the literature on this condition focuses on Asian or White patients [2]. Cutaneous findings in patients with darker skin may be subtle, leading to delayed diagnosis. In this case, the patient's heliotrope rash was diagnosed at an outside facility as eyelid dermatitis. In addition to the classic cutaneous features of DM pertaining to interface dermatitis, certain skin changes are more commonly seen in anti-MDA5 positive DM subtype, some of which may be related to vasculopathy and vasculitis [7]. Though not present in this patient, skin ulcers (such as ulcerated Gottron sign and digital ulcers) are an important diagnostic clue for anti-MDA5 DM and are present in 80%–97% of the patients [1, 7, 8]. Oral ulcers are also more common, with a prevalence of 50% of the patients with anti-MDA5 positive DM compared with 7% with anti-MDA5 negative DM [1]. Though nonscarring alopecia may be present in DM regardless of the myositis-specific antibody, alopecia in anti-MDA5 DM is more often diffuse [7]. In one cohort, panniculitis was present in 20% of the patients with anti-MDA5 DM but was not present in any patients with anti-MDA5 negative DM [1].

Anti-MDA5 DM typically does not present with overt myopathy [9], which can further delay recognition. On physical exam, proximal myopathy is less likely than in anti-MDA5-negative DM [10]. Laboratory findings and muscle biopsy, if performed, typically do not show signs of myositis (as in the current case) [10, 11]. Patients with anti-MDA5 positive DM are more likely to experience arthralgias [10]. For these reasons, awareness of the unique phenotypes associated with anti-MDA5 DM increases the likelihood of diagnosis before rapid pulmonary decline.

In the present case, high-dose methylprednisolone followed by a prednisone and azathioprine taper led to clinical improvement. Interestingly, the patient had already started azathioprine and prednisone 20 mg from an outside facility for a nonspecific myopathy and “eyelid dermatitis” before she developed her pulmonary complications. High-dose glucocorticoids with a second immunosuppressant such as calcineurin inhibitors and/or cyclophosphamide are commonly used to treat anti-MDA5 DM though there have been few randomized controlled trials comparing treatment methods [12, 13]. Changing immunosuppressants, adding tofacitinib, or adding rituximab can be helpful in refractory cases [12, 14]. In patients who do not respond to combined immunosuppressive therapy, IVIG, plasma exchange, or polymyxin B hemoperfusion are considered [13]. Retrospective cohorts have shown IVIG or plasma exchange to reduce mortality by reducing the anti-MDA5 titer or eliminating inflammatory cytokines, respectively [15, 16]. Extracorporeal membrane oxygenation (ECMO) may be required while awaiting therapeutic effect or as a bridge to lung transplantation [13]. For patients such as this one who present to hospitals without the capacity for IVIG or plasma exchange, early initiation of combined immunosuppressive therapy and consideration of transferring to a higher level of care is crucial. The patient had moved to another state after discharge and despite best efforts to contact the patient, she was lost to follow-up.

We describe a case of anti-MDA5 DM with rapidly progressive ILD in the setting of subtle cutaneous findings in a Hispanic woman. Anti-MDA5 DM requires prompt recognition and intervention to reduce the risk of mortality related to respiratory failure. Erythema and other skin changes in DM may be missed in patients of color, necessitating better training in recognizing these eruptions.

## Data Availability

Data sharing not applicable to this article as no datasets were generated or analyzed during the current study.
